# Prognostic value of interim post-treatment SPECT/CT following lutetium-177 (177Lu)-PSMA therapy in patients with metastatic castration-resistant prostate cancer: a systematic review and meta-analysis

**DOI:** 10.3389/fmed.2026.1808563

**Published:** 2026-04-24

**Authors:** Amirreza Shamshirgaran, Mohammad Hadi Samadi, Fatemeh Aboutalebi, Setareh Zahedian, Pegah Sahafi, Michael Saeed, Alessio Rizzo, Giorgio Treglia, Ramin Sadeghi, Atena Aghaee, Emran Askari

**Affiliations:** 1 Urology Research Center, Tehran University of Medical Sciences, Tehran, Iran; 2 Nuclear Medicine Research Center, Mashhad University of Medical Sciences (MUMS), Mashhad, Iran; 3 School of Medicine, Shahid Beheshti University of Medical Sciences, Tehran, Iran; 4 Department of Nuclear Medicine, Candiolo Cancer Institute, FPO-IRCCS, Turin, Italy; 5 Division of Nuclear Medicine, Imaging Institute of Southern Switzerland, Ente Ospedaliero Cantonale, Bellinzona and Lugano, Switzerland; 6 Faculty of Biomedical Sciences, Università della Svizzera Italiana (USI), Lugano, Switzerland; 7 Faculty of Biology and Medicine, University of Lausanne (UNIL), Lausanne, Switzerland

**Keywords:** 177Lu-PSMA therapy, interim imaging, mCRPC, prostate cancer, SPECT/CT

## Abstract

**Background:**

Lutetium-177 (177Lu)-PSMA radioligand therapy (RLT) is established for metastatic castration-resistant prostate cancer (mCRPC), but radiologic monitoring during the treatment lacks standardization. While interim PSMA PET/CT offers accuracy, post-treatment 177Lu-PSMA SPECT/CT provides practical advantages for early response assessment. This systematic review and meta-analysis evaluates the prognostic value of interim post-treatment SPECT/CT parameters on survival outcomes in mCRPC patients.

**Methods:**

Following PRISMA guidelines and PICO framework, PubMed/MEDLINE, Scopus, and Google Scholar were searched up to December 2025 for studies on mCRPC patients receiving 177Lu-PSMA RLT with early post-treatment SPECT/CT. Inclusion required cohort designs reporting hazard ratios (HRs) for overall survival (OS) and PSA-progression-free survival (PSA-PFS). Data extraction included study characteristics, imaging protocols, and outcomes. Quality was assessed using the Oxford CEBM tool. Random-effects meta-analysis pooled HRs; heterogeneity (*I*^2^) and publication bias (funnel plots, Egger’s test, trim-and-fill) were evaluated.

**Results:**

Seven studies (648 patients) were included. Pooled HRs showed emerging new lesions associated with worse OS (HR = 2.78, 95% CI: 1.91–4.06, *p* < 0.001) and PSA-PFS (HR = 4.72, 95% CI: 1.57–14.15, *p* = 0.006). Total tumor volume (TTV) increases predicted inferior OS (HR = 1.80, 95% CI: 1.29–2.50, *p* < 0.001) and PSA-PFS (HR = 2.86, 95% CI: 2.12–3.85, *p* < 0.001). SUVmean and SUVmax reductions were non-significant. Heterogeneity was low-moderate and publication bias had minimal impact post-adjustment.

**Conclusion:**

Interim 177Lu-PSMA SPECT/CT offers prognostic insights, with new lesions and TTV increases signaling poor outcomes. Standardization and prospective validation are needed to integrate it into clinical practice.

## Introduction

Lutetium-177 (^177^Lu)-PSMA therapy is now added to the armamentarium of mCRPC, both in the pre-taxane and post-taxane clinical settings, now paving a way through mHSPC in the lights of encouraging results by PSMAddition and UpFrontPSMA trials (1, 2). However, radiologic monitoring during and after radioligand therapy (RLT) has not been standardized or widely adopted. While for clinical trials, PCWG4 is being prepared, for clinical practice, interim PSMA positron emission tomography with computed tomography (PET/CT) and post-treatment single-photon emission computed tomography combined with computed tomography (SPECT/CT) are two options proposed by different researchers, however, the dominancy of each approach in theranostics centers is not well defined (3).

Using interim PSMA PET/CT is a more accurate strategy since it can capture small (<2 cm) lesions and validated response criteria, such as RECIP (visual or automated) and PSMA PET Progression (PPP), are available for adoption with a better prognostic value as compared to post-treatment PSMA SPECT/CT (4, 5). However, financial issues and difficulty in resource allocation, especially in busy PET centers should be kept in mind. Also, having a concurrent PSMA PET/CT is usually not practical outside trials with intervals from baseline PSMA PET/CT to first PSMA-RLT cycle of 3 months, possibly missing some new lesions occurring within this timeframe which could have downstream consequences. Moreover, from a patient perspective, interim PSMA PET/CT translates to additional set of accommodation, visits, radiotracer to be injected, and radiation safety measures (6, 7).

Using post-treatment ^177^Lu-PSMA SPECT/CT, as an alternative approach, has gained popularity since it makes the clinical workflow and decision making faster, pivoting early ineffective treatment or pausing highly effective treatment, also known as treatment holiday for exceptional responders (7–9). When combined with information gained from PSA responses, SPECT/CT data showed comparable prognostic value as compared to the combined PSA-PSMA PET/CT combination (10). Lastly, post-treatment SPECT/CT could also pave the way for dosimetry purposes.

The response or progression criteria of post-treatment SPECT/CT are not validated yet and the prognostic value of this approach is quite heterogeneous across studies. Herein, we aimed to systematically review the prognostic value of the latter approach in patients with mCRPC, synthesizing evidence on its impact on survival outcomes and clinical decision-making.

## Materials and methods

### Literature search

This systematic review was conducted in accordance with the PRISMA guidelines to ensure transparency and reproducibility. The research question was formulated using the PICO (Population, Intervention, Comparison, Outcome) framework. Population: Patients with mCRPC treated with ^177^Lu-PSMA, Intervention: Post-treatment ^177^Lu-PSMA SPECT/CT imaging, Comparison: other imaging for comparison (if any), and Outcome: the primary outcomes were OS and PFS. The secondary outcomes included any parameter which predict treatment response (such as responders with partial or complete response versus non-responders with stable or progressive disease, based on quantitative metrics like SUV changes, total tumor volume [TTV], or qualitative visual interpretation).

A comprehensive systematic literature search was performed to identify relevant studies evaluating the predictive value of early post-treatment ^177^Lu-PSMA SPECT/CT imaging in patients with mCRPC. The PubMed/MEDLINE and Scopus databases were searched. A complementary search was also conducted in Google to find further studies. The last search was performed in December 2025. The search strategy combined Medical Subject Headings (MeSH) terms and free-text keywords, including but not limited to: (Lu177 OR ^177^Lu OR Lu-177 OR Lutetium) AND SPECT AND prostate AND PSMA. No language restrictions were applied. Additionally, reference lists of included articles and relevant review papers were manually screened for additional eligible studies. Gray literature sources, such as conference abstracts from major oncology meetings (ASCO, ESMO, SNMMI), were searched via Google Scholar and relevant databases to minimize publication bias. The complete, reproducible search strategies for Google Scholar is provided in Supplementary File 1.

### Study selection

Two independent reviewers (FA and SZ) screened titles and abstracts for eligibility, followed by full-text assessment. Disagreements were resolved through consensus or consultation with a third reviewer (AS). Studies were included if they met the following criteria: (1) prospective or retrospective cohort studies involving patients with mCRPC treated with ^177^Lu-PSMA RLT, (2) evaluation of early post-treatment SPECT/CT imaging, (3) reporting of associations between SPECT/CT findings (e.g., response patterns such as complete response, partial response, stable disease, or progressive disease based on quantitative or qualitative metrics like SUV changes or visual interpretation) and clinical outcomes, and (4) provision of sufficient data for extraction, such as hazard ratios (HRs) with 95% confidence intervals (CIs) for survival outcomes.

Exclusion criteria included: (1) case reports with less than 5 patients, reviews, editorials, or animal studies, (2) studies focusing solely on pre-treatment or late post-treatment imaging, (3) investigations without clear linkage between early SPECT/CT results and the specified outcomes, (4) duplicate publications, and (5) studies with overlapping patient cohorts (in which case, the most comprehensive or recent publication was selected).

### Data extraction

Data were extracted independently by two reviewers (FA and SZ) using a standardized form. Extracted variables included: study characteristics (author, year, country, design, sample size), patient demographics (age, PSA levels, prior treatments), imaging protocol details (timing of SPECT/CT, radiotracer dose, interpretation criteria), and outcome measures (HRs with 95% CIs for OS and PFS from multivariable analyses if available, or Kaplan–Meier estimates if not). For survival outcomes, HRs were prioritized from Cox proportional hazards models; if unavailable, they were estimated from Kaplan–Meier curves using Parmar et al. method (11).

### Quality assessment

The methodological quality of included studies was assessed using the Oxford University Centre for Evidence-Based Medicine (CEBM) critical appraisal tool for prognostic studies evaluating the risk of bias of the studies in domains of patient recruitment, follow-up, prognostic factors for outcome, and confounding adjustment. Two independent reviewers (FA and SZ) applied the worksheet to each study. Disagreements were resolved by consensus. Although the QUIPS (Quality in Prognosis Studies) tool is often regarded as the gold standard for domain-specific risk-of-bias assessment in prognostic-factor meta-analyses, the Oxford CEBM tool was chosen for its practicality and applicability to prognostic studies in clinical imaging contexts. The CEBM instrument provided a sufficiently robust and reproducible evaluation for the present review (12).

### Statistical analysis

Pooled HRs with 95% CIs were calculated for various parameters to quantify the predictive association of post-treatment SPECT/CT parameters for OS and PSA-PFS. A random-effects model (DerSimonian-Laird method) was employed to account for anticipated heterogeneity among studies. Heterogeneity was assessed using the Cochrane *Q* test (*p*-value of <0.05 considered as significant) and *I*^2^ statistic (*I*^2^ < 25%: low; 25–50%: moderate; >50%: high). Funnel plots, Egger’s test, and the Duval–Tweedie trim and fill method were used to assess publication bias. Funnel plots depict the standard errors from the selected studies along one axis and their corresponding effect sizes on the other. Any asymmetry observed in these plots may signal possible publication bias, which is assessed through Egger’s regression intercept test (a *p*-value below 0.05 signifies notable bias). The Duval and Tweedie trim-and-fill technique mitigates this bias by progressively eliminating smaller studies to achieve plot symmetry, yielding a revised pooled effect size that helps gauge the extent of publication bias’s impact. Sensitivity and subgroup analyses were performed to explore the influence of heterogeneous definitions of TTV increase (>0% vs. RECIP 1.0 criteria), tracer type, and source of HR (Parmar vs. Kaplan–Meier-derived). All analyses were performed using Comprehensive Meta-Analysis (version 2). The key prognostic indices examined included SUV_max_, SUV_mean_, absolute TTV, reduction in TTV, and emerging new lesion.

## Results

**Figure 1:**
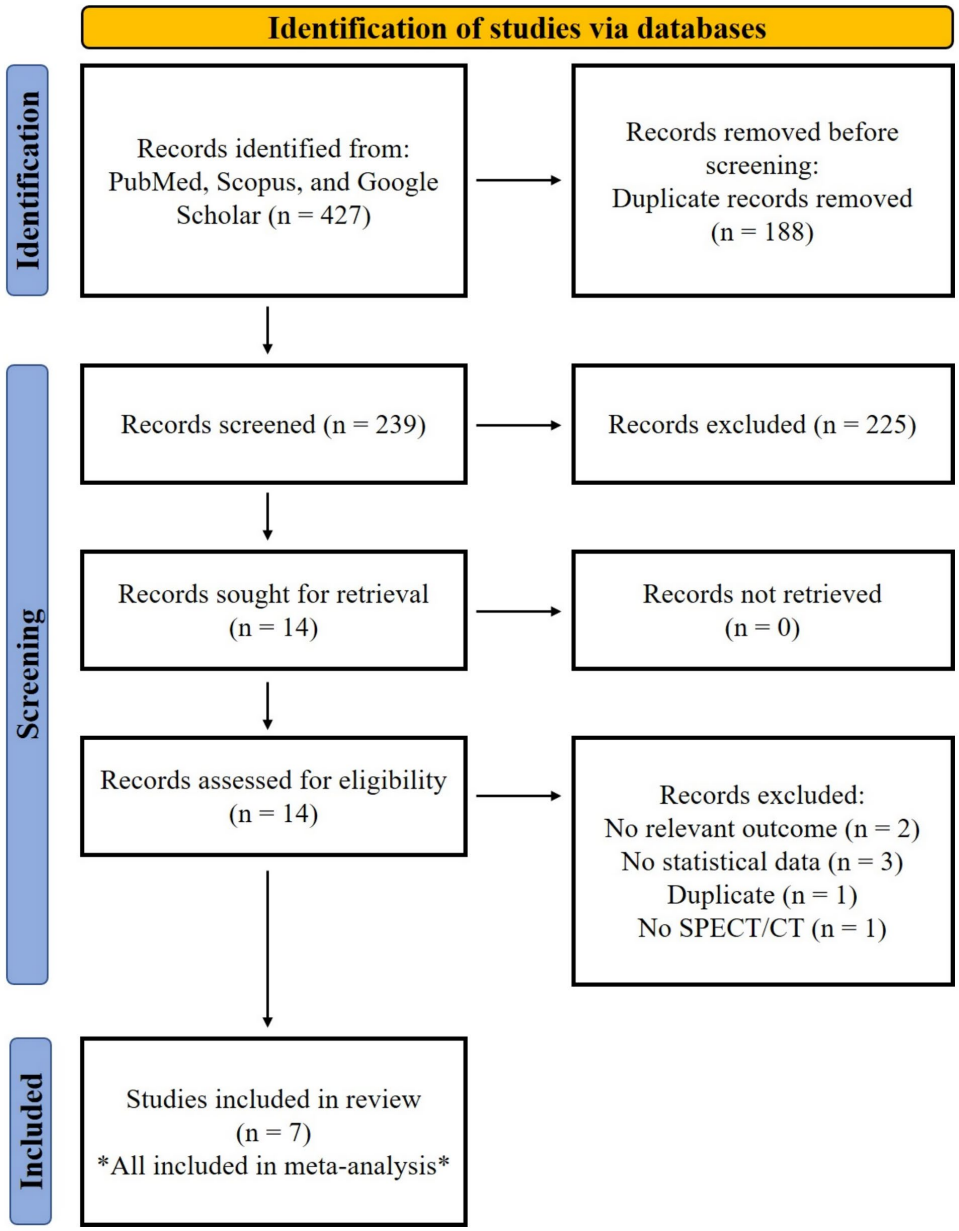
PRISMA flow diagram.

The initial literature search yielded 239 unique records after removal of duplicates. Following title and abstract screening, 14 full-text articles were assessed for eligibility. Of these 2 were excluded: 2 for lacking relevant outcomes (OS or PFS), 3 for focusing lack of statistical data, 1 due to duplication, and 1 for not using SPECT/CT. Ultimately, 7 studies met the inclusion criteria and were included in the meta-analysis. Detailed characteristics of included studies are presented in Table 1. A PRISMA flow diagram detailing the selection process is provided in Figure 1.

**Table 1:** Characteristics of the included articles.

First author	Sample size	SPECT/CT tracer	SPECT/CT timing	Cycle(s)	Parameter	Outcome	HR	95% CI	*p*-value
Demirci (18)	66	^177^Lu-PSMA-617	After 24 h	2	New lesion	OS	7.67	2.76–21.26	<0.001
						PSA-PFS^*^	3.30	0.85–12.68	0.082
Neubauer (13)	73	^177^Lu-PSMA I&T	After 48 h	1 and 2	TTV increase	OS	3.57	1.15–11.04	0.027
						PSA-PFS	1.96	0.95–4.04	0.069
					SUV_mean_	OS	1.02	0.37–3.99	0.730
						PSA-PFS	1.19	0.55–2.56	0.657
					SUV_max_	OS	0.91	0.31–2.65	0.863
						PSA-PFS	1.12	0.56–2.23	0.747
John (14)	127	^177^Lu-PSMA I&T	After 24 h	1 and 2	TTV increase	OS	1.20	0.53–2.70	0.661
						PSA-PFS	2.50	1.49–4.18	<0.001
					SUV_mean_	OS	1.70	0.71–4.06	0.233
						PSA-PFS	2.10	1.18–3.73	0.012
					SUV_max_	OS	1.30	0.60–2.80	0.504
						PSA-PFS	1.80	1.02–3.16	0.042
Pathmanandavel (15)	56	^177^Lu-PSMA-617	After 24 h	1 and 3	TTV increase	OS	1.50	0.57–3.92	0.408
						PSA-PFS	4.10	1.50–11.20	0.006
					SUV_mean_	OS	1.11	0.98–1.25	0.087
						PSA-PFS	1.11	0.99–1.24	0.074
					SUV_max_	OS	1.00	0.99–1.01	0.745
						PSA-PFS	1.00	0.99–1.01	0.577
Unterrainer (16)	105	^177^Lu-PSMA-617	–	2	New lesion	OS	2.70	1.60–4.55	<0.001
					TTV increase	OS	2.00	1.01–3.94	0.046
Kashyap (19)	85	^177^Lu-PSMA-617	–	2	New lesion	OS	2.38	1.35–4.17	0.002
						PSA-PFS	14.90	4.79–46.32	<0.001
Kassas (17)	136	^177^Lu-PSMA I&T	After 24 h	1 and 2	New lesion	OS	2.28	1.37–3.79	0.001
						PSA-PFS	2.59	1.67–4.01	<0.001
					TTV increase	OS	1.83	1.08–3.08	0.023
						PSA-PFS	3.47	2.16–5.55	<0.001

*: PSA-PFS was defined based on the PCWG3 criteria across all included studies (36).

The 7 included studies were published between 2023 and 2025 and comprised prospective or retrospective cohort designs, primarily from single- or multi-center settings in Europe, North America, and Australia. All studies evaluated post-treatment ^177^Lu-PSMA SPECT/CT imaging in patients with mCRPC receiving ^177^Lu-PSMA RLT. ^177^Lu-PSMA I&T and ^177^Lu-PSMA-617 were used in 4 and 3 studies, respectively. Post-treatment SPECT/CT was performed after cycle 2 in three study, after cycle 1 and 2 in three study, and after cycle 1 and 3 in one study. Key predictors assessed included TTV changes, new lesions (NL), SUV metrics, and response categories (such as RECIP 1.0 criteria). Outcomes focused on OS (reported in 7 studies) and PFS (reported in 6 studies). Sample sizes ranged from 56 to 136 patients, with a total of 648 patients across all studies. All studies provided HRs or ORs for survival outcomes, with some including Kaplan–Meier estimates. Heterogeneity in imaging protocols and response criteria was noted, but all included studies used quantitated SPECT/CT mostly 24 h after ^177^Lu-PSMA-617 RLT.

**Figure 2:**
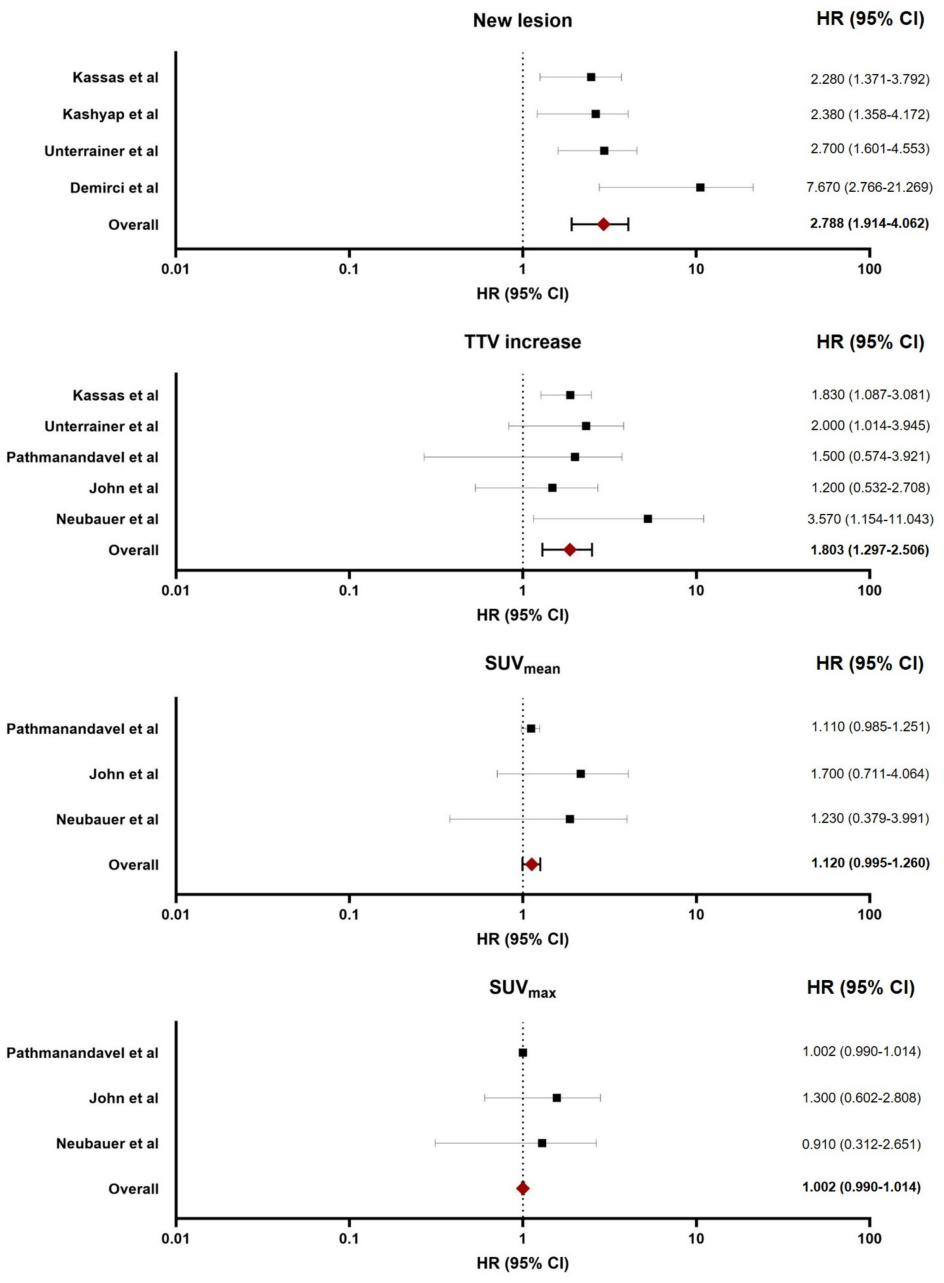
Forest plot illustrating the pooled HR for various post-treatment SPECT/CT regarding OS.

In all included studies, TTV increase and other quantitative parameters were derived exclusively from post-treatment ^177^LU-PSMA SPECT/CT scans and compared serially between interim SPECT/CT time points. There was no direct cross-modality comparison of TTV between baseline diagnostic PET/CT (^68^Ga- or 18F-PSMA) and interim ^177^Lu-SPECT/CT in the prognostic analyses for OS or PSA-PFS. This intra-modality, same-tracer serial approach eliminates the cross-tracer bias (Figure 2).

Definitions of clinically meaningful TTV increase differed among studies. Three studies utilized any increase (>0%) (13–15) and two studies used RECIP 1.0-based categorizations (partial or complete response) (16, 17). Despite this variability, pooled HRs for TTV increase (as a marker of non-response) were calculated using reported data, with inverse thresholds applied where necessary for consistency.

### Quality assessment

Five of the included studies had retrospective design (14, 16–19) while two of them had prospective design (13, 15). All seven studies assembled a defined, representative sample of patients with mCRPC at a common point in the disease course. Follow-up was sufficiently long across studies (median durations ranging from 8.9 to 33.5 months), though completeness was not explicitly reported, with no mention of loss to follow-up or reasons in any study. In five studies, subgroups with differing prognoses were identified and adjusted for important prognostic factors (13, 14, 17–19), while the remaining two lacked such adjustments, increasing confounding risk (15, 16). Applicability to clinical practice was high, as the patient populations were similar to those encountered in routine care, and the evidence could meaningfully impact prognosis. Overall, the included studies demonstrated low risk of bias. Supplementary Table 2 presented the detailed results of quality assessment.

### Prognostic parameters for OS

All seven studies were included in the meta-analysis for OS. The following SPECT/CT parameters were evaluated:

- *Emerging new lesions* (reported in 4 studies): The pooled HR was 2.78 (95% CI: 1.91–4.06, *p*-value < 0.001), indicating a significantly worse OS.
- *TTV increase* (reported in 5 studies): The pooled HR was 1.80 (95% CI: 1.29–2.50, *p*-value < 0.001), indicating significantly inferior OS.
- *SUV_mean_ reduction* (reported in 3 studies): The pooled HR was 1.12 (95% CI: 0.99–1.26, *p*-value = 0.060), which did not reach statistical significance.
- *SUV_max_ reduction* (reported in 3 studies): The pooled HR was 1.00 (95% CI: 0.99–1.01, *p*-value = 0.738), showing no prognostic association.

### Prognostic parameters for PSA-PFS

All seven studies were included in the meta-analysis for PSA-PFS. The following SPECT/CT parameters were evaluated:

- *Emerging new lesions* (reported in 3 studies): The pooled HR was 4.72 (95% CI: 1.57–14.15, *p*-value = 0.006), indicating significantly worse PSA-PFS.
- *TTV increase* (reported in 4 studies): The pooled HR was 2.86 (95% CI: 2.12–3.85, *p*-value < 0.001), indicating significantly inferior PSA-PFS.
- *SUVmean reduction* (reported in 3 studies): The pooled HR was 1.33 (95% CI: 0.88–1.99, *p*-value = 0.165), which did not reach statistical significance.
- *SUVmax reduction* (reported in 3 studies): The pooled HR was 1.17 (95% CI: 0.82–1.66, *p*-value = 0.373), showing no prognostic association.

### Heterogeneity assessment

**Figure 3:**
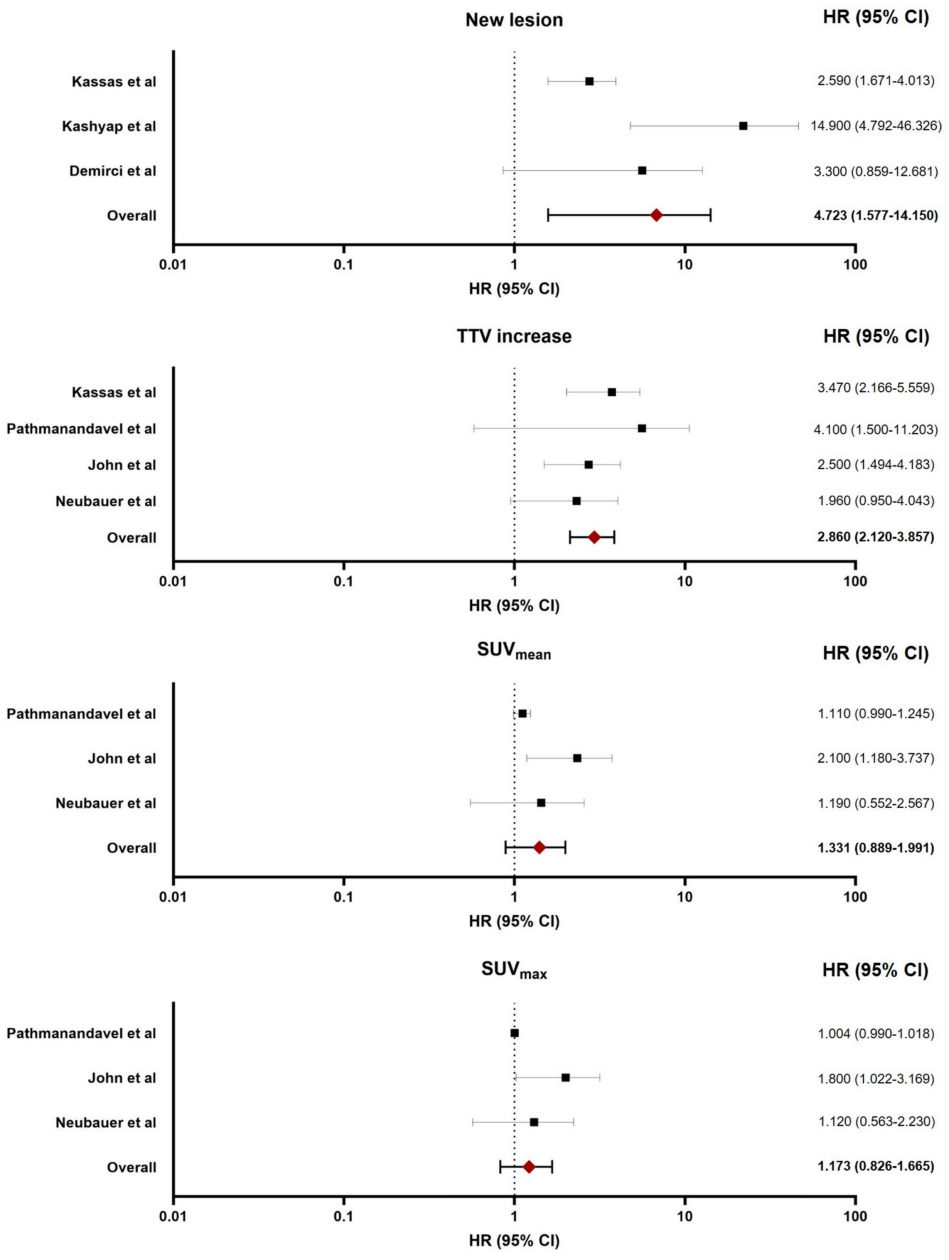
Forest plot illustrating the pooled HR for various post-treatment SPECT/CT regarding PSA-PFS.

For OS, the pooled analyses demonstrated low to moderate heterogeneity for most parameters. Specifically, the emergence of new lesions showed moderate heterogeneity (*I*^2^ = 35.38%, *Q* = 4.64, *p*-value = 0.200), which may stem from differences in study-specific definitions of “new lesions” (such as based on visual interpretation versus quantitative thresholds) or variations in imaging timing across cycles. In contrast, SUV_mean_ reduction, SUV_max_ reduction, and TTV increase exhibited negligible heterogeneity (*I*^2^ = 0% for all, with non-significant *Q*-test *p*-values > 0.05), suggesting consistent effect sizes across studies. This low heterogeneity likely reflects the standardized quantitative nature of these SPECT/CT metrics, which are less susceptible to subjective interpretation and more reproducible in clinical settings (Figure 3).

For PSA-PFS, heterogeneity was more pronounced in several parameters. The analysis for new lesions revealed high heterogeneity (*I*^2^ = 74.85%, Q = 7.95, *p* = 0.019), potentially attributable to inter-study differences in patient populations (varying prior treatment lines or baseline PSA levels) or SPECT/CT protocols (acquisition timing after cycle 1 versus 2). Moderate heterogeneity was observed for SUV_mean_ reduction (*I*^2^ = 55.90%, Q = 4.53, *p* = 0.104) and SUV_max_ reduction (*I*^2^ = 52.20%, Q = 4.18, *p* = 0.123), which could arise from biological variability in PSMA expression during therapy or inconsistencies in SUV calculation methods. TTV increase, however, showed no heterogeneity (*I*^2^ < 0.001%, Q = 2.44, *p* = 0.485), indicating robust consistency and reinforcing its reliability as a prognostic biomarker. Table 2 demonstrated the detailed analysis of heterogeneity.

**Table 2:** The pooled HR of the post-treatment SPECT/CT parameters as prognostic factors for OS and PSA-PFS, using random-effects analysis.

Parameters	Number studies	Effect size and 95% CI			Heterogeneity		
		Pooled HR	95% CI	*p*-value	*Q*-value	*p*-value	*I* ^2^
OS							
New lesion	4	2.78	1.91–4.06	<0.001	4.64	0.200	35.38%
SUV_mean_	3	1.12	0.99–1.26	0.060	0.92	0.629	<0.001%
SUV_max_	3	1.00	0.99–1.01	0.738	0.47	0.791	<0.001%
TTV increase	5	1.80	1.29–2.50	<0.001	2.60	0.627	<0.001%
PSA-PFS							
New lesion	3	4.72	1.57–14.15	0.006	7.95	0.019	74.85%
SUV_mean_	3	1.33	0.88–1.99	0.165	4.53	0.104	55.90%
SUV_max_	3	1.17	0.82–1.66	0.373	4.18	0.123	52.20%
TTV increase	4	2.86	2.12–3.85	<0.001	2.44	0.485	<0.001%

### Sensitivity and subgroup analyses

To address potential clinical heterogeneity in outcome definitions, we conducted the following subgroup analyses:

Subgroup analysis by TTV-increase definition (any increase >0% [(13–15)] vs. RECIP 1.0-based progression [(16, 17)]): Pooled HR for OS remained 1.69 (95% CI: 0.92–3.09) and 1.89 (95% CI: 1.25–2.85), respectively; for PSA-PFS, 2.51 (95% CI: 1.70–3.69) and 3.47 (95% CI: 2.16–5.55). No significant between-subgroup difference for OS and PSA-PFA (*p*-value = 0.765 and 0.298 respectively).

Leave-one-out sensitivity analysis for new lesions on PSA-PFS to explore the heterogeneity sources (cycle timing, tracer type, Kaplan–Meier-derived HRs, or prospective vs. retrospective design). Although all included studies evaluating new lesions were retrospective and performed SPECT/CT after cycle 2 (eliminating study design and cycle timing as sources of variation), we conducted leave-one-out sensitivity analysis stratified by tracer type (PSMA-617 vs. I&T) and extracted HRs methods (Parmar method [in Kashyap et al. study] vs., multivariable Cox). After excluding the study that used ^177^Lu-PSMA I&T, heterogeneity decreased to *I*^2^ = 64.49% (*p*-value = 0.093), which remains moderately high but no longer statistically significant at the conventional threshold. Importantly, the pooled HR remained robust and statistically significant at 7.33 (95% CI: 1.67–32.06, *p*-value = 0.008). However, after excluding estimated HRs, heterogeneity decreased to *I*^2^ < 0.001 (*p*-value = 0.737), showing that HRs extraction method was consider as a heterogeneity source. This time also the pooled HR remained robust and significant (HR = 2.65 (95% CI: 1.74–4.02, *p*-value < 0.001).

Sensitivity analysis excluding studies with Kaplan–Meier-derived HRs were also conducted for other parameters. Only 2 other parameters (new lesion and TTV increase for OS in Unterrainer et al. study) required HR estimation via the Parmar method. The remainder provided direct multivariable Cox HRs. Sensitivity analyses yielded virtually identical pooled estimates (TTV increase for OS: HR = 1.74, 95% CI: 1.19–2.54, *p*-value = 0.004; new lesion for OS: HR = 2.98, 95% CI: 1.67–5.31, *p*-value < 0.001), confirming the robustness of pooled HRs.

### Publication bias

**Figure 4:**
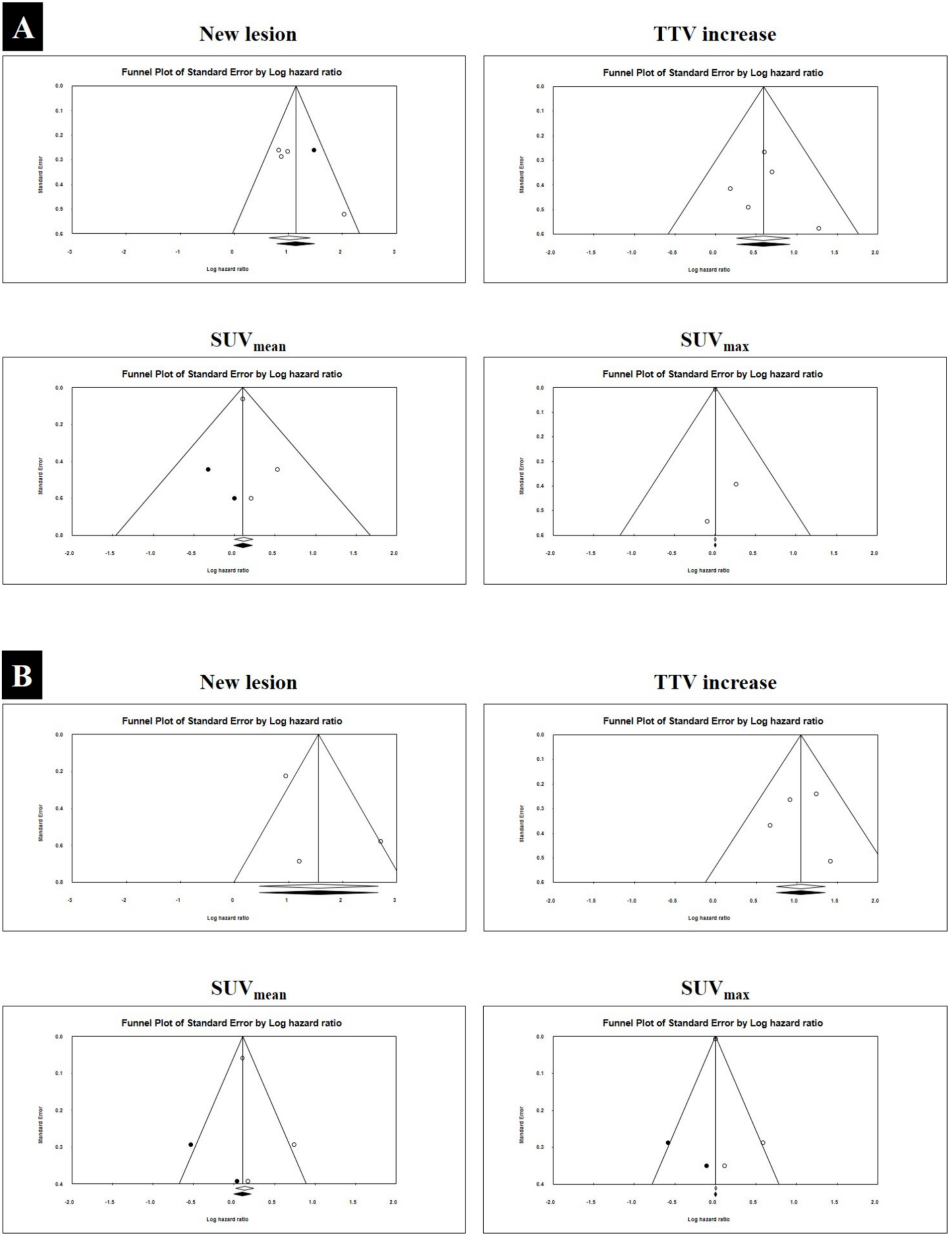
The funnel plot for HR pooling for SPECT/CT parameters regarding OS **(A)** and PSA-PFS **(B)**. The white circles depict the studies that were incorporated, whereas the white diamond signifies the pooled HR derived from these studies. The black circles denote the studies that were trimmed to rectify asymmetry. On the other hand, the black diamond represents the modified pooled HR that adjusts for possible publication bias, computed through the Duval-Tweedie trim and fill method.

Publication bias for OS prognostic parameters was assessed through visual inspection of funnel plots (Figure 4A) and Egger’s regression test. Funnel plot asymmetry was noted for the emergence of new lesions and SUV_mean_ reduction, with Egger’s regression intercepts of 4.45 (*p*-value = 0.034) and 0.62 (*p*-value = 0.405), respectively. Application of the Duval and Tweedie trim-and-fill method imputed two missing studies for each parameter, resulting in symmetrical funnel plots. The adjusted pooled HRs were 3.03 (95% CI: 2.35–3.91) for new lesions and 1.11 (95% CI: 0.98–1.24) for SUV_mean_ reduction.

Similarly, potential publication bias for PSA-PFS parameters was evaluated using funnel plots (Figure 4B) and Egger’s regression test. Asymmetry was observed for SUV_mean_ and SUV_max_ reduction, with intercepts of 1.36 (*p* = 0.479) and 1.19 (*p* = 0.404), respectively. The trim-and-fill method imputed two studies per parameter to restore symmetry, yielding adjusted pooled HRs of 1.11 (95% CI: 0.99–1.23) for SUV_mean_ reduction and 1.00 (95% CI: 0.99–1.01) for SUV_max_ reduction. These adjustments suggest that publication bias, where present, had minimal impact on the overall findings.

## Discussion

While interim PSMA PET/CT has traditionally served as the reference standard for PSMA-based response assessment, the practicality, accessibility, and increasingly validated prognostic value of mid-treatment ^177^Lu-PSMA SPECT/CT position it as a promising alternative for routine cycle-integrated response monitoring. The findings of this systematic review further consolidate the role of SPECT-derived biomarkers and highlight their significance in contemporary theranostic practice.

The results of this systematic review and meta-analysis underscore the prognostic utility of interim post-treatment ^177^Lu-PSMA SPECT/CT in patients with mCRPC undergoing ^177^Lu-PSMA RLT. Across the seven included studies encompassing 648 patients, the emergence of new lesions and increases in TTV, emerged as robust predictors of inferior OS and PSA-PFS. Specifically, the pooled HRs indicated that the presence of new lesions was associated with a 2.78-fold increased risk of death (95% CI: 1.91–4.06, *p*-value < 0.001) and a 4.72-fold increased risk of PSA progression (95% CI: 1.57–14.15, *p*-value = 0.006). Similarly, TTV increases correlated with HRs of 1.80 for OS (95% CI: 1.29–2.50, *p*-value < 0.001) and 2.86 for PSA-PFS (95% CI: 2.12–3.85, *p*-value < 0.001). These findings align with the growing recognition of post-treatment SPECT/CT as a practical, non-invasive tool for early response assessment, enabling timely adjustments in therapeutic strategies, such as treatment escalation or de-escalation (20).

The prognostic significance of new lesions and TTV changes highlights the value of ^177^Lu-PSMA SPECT/CT in capturing disease dynamics during therapy. Among these factors, the detection of new lesions appears particularly impactful, as it often precedes biochemical progression and signals treatment resistance in up to 50% of cases, as noted in prior reports (21, 22). This emphasis on new lesions is reflected in the evolving PCWG4 guidelines, which prioritize such indicators for standardizing response evaluation in clinical trials (3). In contrast, while TTV increases also demonstrate strong prognostic relevance, their interpretation can vary based on thresholds, underscoring the need for consensus definitions. Recent comparative studies have suggested that PSMA PET/CT may offer superior accuracy over combined computed tomography/bone scan (CT/BS) modalities for prognosticating outcomes in mCRPC, potentially due to better detection of bone-dominant disease (23). However, direct head-to-head comparisons between ^177^Lu-PSMA SPECT/CT and CT/BS are lacking, limiting our ability to position SPECT/CT within the broader imaging landscape. Furthermore, optimized criteria for response assessment using ^177^Lu-PSMA SPECT/CT remain elusive. A recent study analyzing data from the Enza-P trial demonstrated that quantitative TTV metrics outperformed visual image interpretation in predicting treatment response, suggesting that volumetric approaches could enhance the reliability of SPECT/CT over subjective assessments (24). As TTV change and new lesion detection were assessed serially on 177Lu-PSMA SPECT/CT only, potential variability in baseline PET tracers and associated benign bone uptake did not introduce heterogeneity into the pooled prognostic estimates.

Variability in SPECT/CT acquisition timing (24 h versus 48 h post-injection) across studies represents a potential source of heterogeneity for quantitative metric. Tumor uptake and background clearance on 177Lu-PSMA SPECT/CT evolve over time, with improved tumor-to-background ratios typically observed at later imaging points (24–48 h). While most included studies performed scans at 24 h, one used 48 h. As mentioned before, TTV change and new lesion detection were evaluated as relative changes on serially acquired scans using the same intra-study timing protocol, the effect on these binary or directional prognostic parameters is likely modest. In contrast, absolute SUVmax and SUVmean values are more timing-dependent and may be influenced by ongoing clearance and redistribution, potentially attenuating their prognostic signal (25, 26).

These observations are somehow consistent with evidence from larger studies employing baseline ^68^Ga-PSMA PET/CT parameters, where similar prognostic factors have been validated. In landmark trials like VISION and TheraP, new lesions and TTV changes on baseline PET scans were strongly associated with survival outcomes, with HRs for OS and PFS in comparable ranges (27, 28). The Enza-P study further reinforced the prognostic role of TTV in the context of PSMA-targeted therapies, showing its utility in differentiating responders from non-responders (27). The alignment between our SPECT/CT-derived HRs and these PET-based findings suggests that post-treatment ^177^Lu-PSMA SPECT/CT may serve as a practical, accessible, and potentially more cost-effective alternative to PET/CT, particularly in resource-constrained settings where PET availability is limited (26, 29). This is particularly relevant given the logistical advantages of SPECT/CT, including its integration into routine post-therapy workflows without additional radiotracer injections or patient visits.

Notably, changes in standardized uptake value metrics, such as SUV_mean_ and SUV_max_ reductions, did not demonstrate significant prognostic value in our analysis (pooled HRs of 1.12 and 1.00 for OS respectively, *p*-value > 0.05). Differences in SPECT/CT scanner technology, reconstruction algorithms, and lack of cross-center harmonization may have further reduced the reliability of per-lesion SUV metrics, contributing to their failure to reach statistical significance, whereas volumetric TTV and visual new-lesion assessments appear more robust to such technical variability. This contrasts with baseline imaging data from trials like VISION and TheraP, where SUV_mean_ on pre-treatment ^68^Ga-PSMA PET scans was a significant predictor of response and survival (30, 31). The discrepancy may stem from differences in radiopharmaceuticals and imaging modalities. ^177^Lu-based SPECT/CT, which relies on therapeutic lutetium emissions, inherently provides lower resolution and quantitative accuracy compared to diagnostic ^68^Ga-PSMA PET/CT (32). Factors such as partial volume effects, variable tumor uptake kinetics, and the therapeutic dose’s influence on post-treatment scans could further attenuate SUV reliability (33). Emerging metrics, such as the total lesion quotient (TLQ, defined as TTV divided by SUV_mean_), have shown promise in recent PET studies, where TLQ exhibited the strongest association with OS prediction (34). However, to our knowledge, no studies have yet evaluated TLQ specifically in the context of ^177^Lu-PSMA SPECT/CT scans, representing a gap that warrants investigation to potentially refine prognostic models.

Beyond individual parameters, the potential added value of serial or continuous post-treatment SPECT/CT assessments remains underexplored. The cumulative impact of sequential TTV reductions across multiple cycles (e.g., after cycles 1, 2, and 3) could provide incremental prognostic insights, such as identifying exceptional responders eligible for treatment holidays. None of the included studies systematically quantified this, highlighting an opportunity for future research to evaluate dynamic imaging trajectories and their correlation with long-term outcomes.

Finally, our meta-analysis has several limitations. The protocol for this systematic review and meta-analysis was not prospectively registered in PROSPERO or a similar database. Although the study adhered strictly to PRISMA 2020 guidelines and the pre-defined PICO framework, the absence of registration may introduce a minor risk of reporting bias. The evidence base is constrained by a limited number of studies (*n* = 7) and modest sample sizes (median 85 patients per study), which may reduce generalizability. Most studies were retrospective, introducing risks of selection bias and incomplete follow-up data, although quality assessments indicated overall low bias. Variability in TTV contouring methods was another limitation across studies. Differences in exact thresholds, manual correction procedures, and scanner-specific reconstruction could influence absolute volume measurements. Although TTV increase was evaluated as relative interval change on serial same-modality SPECT/CT (which reduces the impact of absolute threshold differences) and showed negligible heterogeneity (*I*^2^ < 0.001%), this methodological variability remains a potential source of bias that warrants standardization in future prospective studies. Heterogeneity was evident in some analyses, particularly for PSA-PFS parameters like new lesions (*I*^2^ = 74.85%), attributable to variations in imaging timing (such as after cycle 1 vs. 2), response criteria, and patient characteristics. Publication bias was detected in funnel plots for certain variables, though trim-and-fill adjustments yielded minimal changes in pooled HRs, suggesting negligible impact on conclusions. Additionally, given the small number of studies (*n* = 7 overall, 3–5 per parameter), the statistical power of Egger’s test for funnel-plot asymmetry is known to be low. However, visual inspection and trim-and-fill adjustment provided reassurance that any potential bias had negligible influence on the pooled estimates (35). Future prospective, multicenter studies with standardized protocols are essential to validate these findings and address these limitations.

## Conclusion

Current evidence supports that mid-treatment ^177^Lu-PSMA SPECT/CT provides valuable prognostic information in mCRPC patients. Particularly, the emergence of new lesions and increases in TTV are associated with worse survival outcomes. Changes in per-lesion uptake (SUV_max_ or SUV_mean_) alone appear to have limited prognostic value. While aligned with PET/CT evidence, the modality’s unique advantages and limitations call for further refinement, including exploration of novel metrics like TLQ and serial assessments, to optimize its role in personalized theranostics. Standardization of imaging protocols and prospective validation are needed. Incorporating such imaging biomarkers into clinical decision-making could optimize therapeutic strategies and improve patient outcomes.

## Data Availability

The original contributions presented in the study are included in the article/Supplementary material, further inquiries can be directed to the corresponding author.

## References

[1] Tagawa ST Sartor O Piulats JM Saad F Fizazi K Reid AHM . LBA6 phase III trial of [177Lu]Lu-PSMA-617 combined with ADT + ARPI in patients with PSMA-positive metastatic hormone-sensitive prostate cancer (PSMAddition). Ann Oncol. (2025) 36:S1627–8. doi: 10.1016/j.annonc.2025.09.101

[2] Azad AA Bressel M Tan H Voskoboynik M Suder A Weickhardt AJ . Sequential [177Lu]Lu-PSMA-617 and docetaxel versus docetaxel in patients with metastatic hormone-sensitive prostate cancer (UpFrontPSMA): a multicentre, open-label, randomised, phase 2 study. Lancet Oncol. (2024) 25:1267–76. doi: 10.1016/S1470-2045(24)00440-6, 39293461

[3] Hofman MS Gafita A Bressel M Alipour R Levy S Emmett L . 1608P prostate cancer working group 4 (PCWG4) preliminary criteria using serial PSMA PET/CT for response evaluation: analysis from the PRINCE trial. Ann Oncol. (2024) 35:S970. doi: 10.1016/j.annonc.2024.08.1689

[4] Gafita A Djaileb L Rauscher I Fendler WP Hadaschik B Rowe SP . Response evaluation criteria in PSMA PET/CT (RECIP 1.0) in metastatic castration-resistant prostate Cancer. Radiology. (2023) 308:e222148. doi: 10.1148/radiol.222148, 37432081 PMC10374938

[5] Fanti S Hadaschik B Herrmann K. Proposal for systemic-therapy response-assessment criteria at the time of PSMA PET/CT imaging: the PSMA PET progression criteria. J Nucl Med. (2020) 61:678–82. doi: 10.2967/jnumed.119.233817, 31806774 PMC7198387

[6] Berger M Gould MK Barnett PG. The cost of positron emission tomography in six United States veterans affairs hospitals and two academic medical Centers. Am J Roentgenol. (2003) 181:359–65. doi: 10.2214/ajr.181.2.1810359, 12876011

[7] Fanti S Goffin K Hadaschik BA Herrmann K Maurer T MacLennan S . Consensus statements on PSMA PET/CT response assessment criteria in prostate cancer. Eur J Nucl Med Mol Imaging. (2021) 48:469–76. doi: 10.1007/s00259-020-04934-4, 32617640 PMC7835167

[8] Holzgreve A Delker A Ells Z Brosch-Lenz J Unterrainer LM Nikitas J . Randomized phase 2 trial of an extended and flexible dosing schedule of (177)Lu-PSMA molecular radiotherapy in patients with metastatic castration-resistant prostate Cancer (FLEX-MRT): study protocol. J Nucl Med. (2025) 66:1639–45. doi: 10.2967/jnumed.125.269495, 40876952 PMC12487728

[9] Samadi MH Forouzanian S Sahafi P Jafari Zarrin Ghabaei F Aghaee A. Sudden diffuse hepatic metastases after first 177Lu-PSMA cycle in bone predominant mCRPC patient. Clin Nucl Med.(2022) 10. doi: 10.1097/RLU.0000000000006170 42247620

[10] Kim J Lee S Kim D Kim HJ Oh KT Kim SJ . Combination of [(18)F]FDG and [(18)F]PSMA-1007 PET/CT predicts tumour aggressiveness at staging and biochemical failure postoperatively in patients with prostate cancer. Eur J Nucl Med Mol Imaging. (2024) 51:1763–72. doi: 10.1007/s00259-023-06585-7, 38200396

[11] Parmar MK Torri V Stewart L. Extracting summary statistics to perform meta-analyses of the published literature for survival endpoints. Stat Med. (1998) 17:2815–34. doi: 10.1002/(SICI)1097-0258(19981230)17:24<2815::AID-SIM110>3.0.CO;2-8, 9921604

[12] Hayden JA van der Windt DA Cartwright JL Côté P Bombardier C. Assessing bias in studies of prognostic factors. Ann Intern Med. (2013) 158:280–6. doi: 10.7326/0003-4819-158-4-201302190-00009 23420236

[13] Neubauer MC Nicolas GP Bauman A Fani M Nitzsche E Afshar-Oromieh A . Early response monitoring during [177Lu]Lu-PSMA I&T therapy with quantitated SPECT/CT predicts overall survival of mCRPC patients: subgroup analysis of a Swiss-wide prospective registry study. Eur J Nucl Med Mol Imaging. (2024) 51:1185–93. doi: 10.1007/s00259-023-06536-2, 38038755 PMC10881597

[14] John N Pathmanandavel S Crumbaker M Counter W Ho B Yam AO . (177)Lu-PSMA SPECT quantitation at 6 weeks (dose 2) predicts short progression-free survival for patients undergoing (177)Lu-PSMA-I&T therapy. J Nucl Med. (2023) 64:410–5. doi: 10.2967/jnumed.122.264677, 36215568

[15] Pathmanandavel S Crumbaker M Ho B Yam AO Wilson P Niman R . Evaluation of (177)Lu-PSMA-617 SPECT/CT quantitation as a response biomarker within a prospective (177)Lu-PSMA-617 and NOX66 combination trial (LuPIN). J Nucl Med. (2023) 64:221–6. doi: 10.2967/jnumed.122.264398, 36008120 PMC9902857

[16] Unterrainer LM de Leiris N Unterrainer M Delker A Hempel L Ells Z . Evidence-based clinical protocols to monitor efficacy of [(177)Lu]Lu-PSMA radiopharmaceutical therapy in metastatic castration-resistant prostate cancer using real-world data. J Nucl Med. (2025) 66:1054–60. doi: 10.2967/jnumed.124.269431, 40274370

[17] Kassas M Devriendt L Manley M Guiot T Marin C Danieli R . [177Lu]Lu-PSMA SPECT/CT for early response assessment using quantitative RECIP 1.0. Eur J Nucl Med Mol Imaging. (2025) 53:2257–70. doi: 10.1007/s00259-025-07613-4, 41207904

[18] Demirci RA Gulati R Hawley JE Yezefski T Haffner MC Cheng HH . SPECT/CT in early response assessment of patients with metastatic castration-resistant prostate cancer receiving (177)Lu-PSMA-617. J Nucl Med. (2024) 65:1945–51. doi: 10.2967/jnumed.124.267665, 39510589 PMC11937724

[19] Kashyap R Buteau JP Bressel M Eifer M Bollampally N Jackson P . Prognostic value of posttherapy SPECT/CT for overall survival in patients undergoing [(177)Lu]Lu-PSMA-617 radiopharmaceutical therapy: results from 3 clinical trials. J Nucl Med. (2025) 66:1265–70. doi: 10.2967/jnumed.125.269640, 40610228 PMC13670944

[20] Alkahtani TO. Investigating the significance of SPECT/CT-SUV for monitoring 177Lu-PSMA-targeted radionuclide therapy: a systematic review. BMC Med Imaging. (2025) 25:28. doi: 10.1186/s12880-025-01571-x, 39875849 PMC11776189

[21] Prasad V Huang K Prasad S Makowski MR Czech N Brenner W. In comparison to PSA, interim ga-68-PSMA PET/CT response evaluation based on modified RECIST 1.1 after 2(nd) cycle is better predictor of overall survival of prostate cancer patients treated with (177)Lu-PSMA. Front Oncol. (2021) 11:578093. doi: 10.3389/fonc.2021.578093, 33816225 PMC8010239

[22] Han S Woo S Kim YI Lee JL Wibmer AG Schoder H . Concordance between response assessment using prostate-specific membrane antigen PET and serum prostate-specific antigen levels after systemic treatment in patients with metastatic castration resistant prostate cancer: a systematic review and Meta-analysis. Diagnostics (Basel). (2021) 11. doi: 10.3390/diagnostics11040663, 33917006 PMC8067707

[23] Lengana T Lawal IO Boshomane TG Popoola GO Mokoala KMG Moshokoa E . (68)ga-PSMA PET/CT replacing bone scan in the initial staging of skeletal metastasis in prostate Cancer: a fait accompli? Clin Genitourin Cancer. (2018) 16:392–401. doi: 10.1016/j.clgc.2018.07.009, 30120038

[24] Emmett L Papa N Subramaniam S Crumbaker M Nguyen A Joshua AM . Prognostic and predictive value of baseline PSMA-PET total tumour volume and SUVmean in metastatic castration-resistant prostate cancer in ENZA-p (ANZUP1901): a substudy from a multicentre, open-label, randomised, phase 2 trial. Lancet Oncol. (2025) 26:1168–77. doi: 10.1016/S1470-2045(25)00339-0, 40752515

[25] Swiha M Pathmanandavel S Papa N Sabahi Z Li S Zheng A . Comparison of posttherapy 4- and 24-hour [(177)Lu]Lu-PSMA SPECT/CT and pretherapy PSMA PET/CT in assessment of disease in men with metastatic castration-resistant prostate Cancer. J Nucl Med. (2024) 65:1939–44. doi: 10.2967/jnumed.124.267606, 39477497

[26] Uribe C Iravani A Savir-Baruch B Jacene H Graves SA Dewaraja YK . Summary: SNMMI/ACNM procedure standard for posttreatment imaging of (177)Lu-based radiopharmaceuticals. J Nucl Med. (2025) 66:1528–37. doi: 10.2967/jnumed.125.270979, 40935610

[27] Emmett L Subramaniam S Crumbaker M Nguyen A Joshua AM Weickhardt A . [177Lu]Lu-PSMA-617 plus enzalutamide in patients with metastatic castration-resistant prostate cancer (ENZA-p): an open-label, multicentre, randomised, phase 2 trial. Lancet Oncol. (2024) 25:563–71. doi: 10.1016/S1470-2045(24)00135-9, 38621400

[28] Sartor O de Bono J Chi KN Fizazi K Herrmann K Rahbar K . Lutetium-177–PSMA-617 for metastatic castration-resistant prostate Cancer. N Engl J Med. (2021) 385:1091–103. doi: 10.1056/NEJMoa2107322, 34161051 PMC8446332

[29] Albalooshi B al Sharhan M Bagheri F Miyanath S Ray B Muhasin M . Direct comparison of (99m)Tc-PSMA SPECT/CT and (68)Ga-PSMA PET/CT in patients with prostate cancer. Asia Ocean J Nucl Med Biol. (2020) 8:1–7. doi: 10.22038/aojnmb.2019.43943.1293, 32064277 PMC6994779

[30] Kuo PH Morris MJ Hesterman J Kendi AT Rahbar K Wei XX . Quantitative (68)Ga-PSMA-11 PET and clinical outcomes in metastatic castration-resistant prostate Cancer following (177)Lu-PSMA-617 (VISION trial). Radiology. (2024) 312:e233460. doi: 10.1148/radiol.233460, 39162634 PMC11366674

[31] Buteau JP Martin AJ Emmett L Iravani A Sandhu S Joshua AM . PSMA and FDG-PET as predictive and prognostic biomarkers in patients given [(177)Lu]Lu-PSMA-617 versus cabazitaxel for metastatic castration-resistant prostate cancer (TheraP): a biomarker analysis from a randomised, open-label, phase 2 trial. Lancet Oncol. (2022) 23:1389–97. doi: 10.1016/S1470-2045(22)00605-2, 36261050

[32] Kratochwil C Giesel FL Stefanova M Benešová M Bronzel M Afshar-Oromieh A . PSMA-targeted radionuclide therapy of metastatic castration-resistant prostate Cancer with177Lu-Labeled PSMA-617. J Nucl Med. (2016) 57:1170–6. doi: 10.2967/jnumed.115.171397 26985056

[33] Boellaard R Delgado-Bolton R Oyen WJG Giammarile F Tatsch K Eschner W . FDG PET/CT: EANM procedure guidelines for tumour imaging: version 2.0. Eur J Nucl Med Mol Imaging. (2015) 42:328–54. doi: 10.1007/s00259-014-2961-x, 25452219 PMC4315529

[34] Kimura K Murthy V Voter A Yadav S Theus L Nguyen A . Evaluation of PSMA PET/CT derived predictors for treatment response to [177Lu]Lu-PSMA-617: results from the U.S. expanded-access program. J Nucl Med. (2025) 66:251307 10.2967/jnumed.125.270789 PMC13138095 41679922

[35] Sterne JAC Sutton AJ Ioannidis JPA Terrin N Jones DR Lau J . Recommendations for examining and interpreting funnel plot asymmetry in meta-analyses of randomised controlled trials. BMJ. (2011) 343:d4002. doi: 10.1136/bmj.d4002, 21784880

[36] Scher HI Morris MJ Stadler WM Higano C Basch E Fizazi K . Trial design and objectives for castration-resistant prostate cancer: updated recommendations from the prostate Cancer clinical trials working group 3. J Clin Oncol. (2016) 34:1402–18. doi: 10.1200/JCO.2015.64.2702, 26903579 PMC4872347

