# Supplementary material for: Prognostic value of interim post-treatment SPECT/CT following lutetium-177 (177Lu)-PSMA therapy in patients with metastatic castration-resistant prostate cancer: a systematic review and meta-analysis
Source: Front Med (Lausanne). 2026 Apr 24;13:1808563. doi: 10.3389/fmed.2026.1808563 (PMC13152746; doi:10.3389/fmed.2026.1808563)
Supplement: Supplementary file 1 [file Table_1.docx]

**Supplementary Table 1.** Risk-of-bias of the included studies using the Oxford University for CEBM checklist for Prognostic Studies.

| **Author** | **1. Patient Recruitment** | **2. Follow-up** | **3. Recurrence Assessment** | **4. Prognostic Factors** | **5. Adjustment for confounding** |
| --- | --- | --- | --- | --- | --- |
| Demirci et al. | Retrospective single-center study; 66 consecutive mCRPC patients who received ≥2 LuPSMA cycles. | Median follow-up from cycle 2: 42 weeks. No loss to follow-up mentioned. | Disease progression assessed via new lesions (PSMA-avid or non-avid) on SPECT/CT. | TTV, new lesions. | Yes (multivariate Cox regression adjusted for changes in PSA). |
| Neubauer et al. | Prospective Swiss national registry subgroup (observational); 73 included with ≥2 cycles of [177Lu]Lu-PSMA I&T. | Median follow-up: 8.9 months. No loss to follow-up mentioned. | Disease progression assessed via PSA-PFS and OS. | Changes in TTV, PSA, SUVmax, SUVmean. | Yes (univariate and multivariate Cox with all possible predictors and confounding factors). |
| John et al. | Retrospective single-center registry; 127 men with progressive mCRPC; screening PSMA PET/CT (SUVmax >15 at one site, >10 at all measurable sites). | Not reporting overall follow-up; Median PSA PFS: 6.1 months; median OS: 16.8 months. No loss to follow-up mentioned. | Disease progression assessed via PSA PFS; response via changes in SPECT TTV. | Increase in SPECT TTV (>20% or any increase), SUVmax, SUVmean. | Yes (univariable and multivariable Cox proportional-hazards regression). |
| Pathmanandavel et al. | Prospective single-center phase I/II trial; 56 patients enrolled; screening with FDG/PSMA PET/CT, bone scan, CT. | Not reporting overall follow-up; Median PSA-PFS: 6.3 months; median OS: 12.3 months. No loss to follow-up mentioned. | Disease progression assessed via PSA PFS; response via changes in SPECT TTV. | Changes in SPECT TTV (>30% increase), SUVmax, SUVmean; PSA progression at week 12. | No (small cohort; no multivariable analysis mentioned). |
| Unterrainer et al. | Retrospective multicenter study; 105 patients with mCRPC who received ≥2 LuPSMA cycles | Not reporting overall follow-up; Median OS: 12.3 months; No loss to follow-up mentioned. | Disease progression assessed via RECIP 1.0 (visual) on SPECT/CT; PSA. | Progressive disease by RECIP-PD, new lesions, changes in PSMA-VOL; PSA + RECIP. | No (univariate Cox regression; no explicit multivariable adjustment for confounders mentioned). |
| Kashyap et al. | Retrospective cohort from 3 clinical trials; 89 patients screened; ≥12 months follow-up. | Median follow-up: 33.5 months. No loss to follow-up mentioned. | Disease progression assessed via PSA progression, new PSMA-avid metastases on cycle 2 SPECT/CT, and OS. | Changes in TTV; PSA; appearance of new lesions on SPECT/CT. | Yes (multivariable Cox models adjusted for age, Gleason score, and change in PSA). |
| Kassas et al. | Retrospective single-center study; 136 mCRPC patients treated with ≥2 cycles of [177Lu]Lu-PSMA-I&T. | Median follow-up: 23 months. No loss to follow-up mentioned. | Disease progression assessed via quantitative RECIP 1.0 on SPECT/CT. | Increase in SUVmean, TTV increase ≥20%, new lesions; RECIP-PD; baseline factors. | Yes (uni- and multivariable Cox regression; models adjusted for collinearity and clinical variables like baseline ALP, hemoglobin). |
