# Supplementary material for: Prognostic value of interim post-treatment SPECT/CT following lutetium-177 (177Lu)-PSMA therapy in patients with metastatic castration-resistant prostate cancer: a systematic review and meta-analysis
Source: Front Med (Lausanne). 2026 Apr 24;13:1808563. doi: 10.3389/fmed.2026.1808563 (PMC13152746; doi:10.3389/fmed.2026.1808563)
Supplement: Supplementary file 2 [file Table_2.docx]

**Detailed Search Strategies for Google Scholar**

“177Lu PSMA” OR “Lutetium-177 PSMA” OR “Lu177 PSMA” OR “Lu-177 PSMA” OR “177Lu-PSMA” SPECT (interim OR “post-treatment” OR “post therapy” OR “early response” OR “response assessment”) (mCRPC OR “metastatic castration-resistant prostate cancer” OR prostate)

Date filter applied: publications up to 31 December 2025

The first 200 results were manually screened by two independent reviewers.

Additional hand-searching was performed on reference lists of included articles and relevant review papers, as well as conference abstracts from ASCO, ESMO, and SNMMI.
